# DNA Methylation and mRNA Expression of SEPT9 and AKR1B1 in Ovarian Cancer: Diagnostic and Prognostic Implications

**DOI:** 10.3390/ijms27146336

**Published:** 2026-07-16

**Authors:** Mateusz Kozłowski, Dominika Borzyszkowska, Michał Lubkowski, Nina Komaniecka, Aleksandra Mikulka, Radosław Birger, Piotr Kolczewski, Agnieszka Brodowska, Mateusz Kurzawski, Agnieszka Kempinska-Podhorodecka, Aneta Cymbaluk-Płoska

**Affiliations:** 1 Department of Gynecological Surgery and Gynecological Oncology of Adults and Adolescents, Pomeranian Medical University in Szczecin, Al. Powstańców Wielkopolskich 72, 70-111 Szczecin, Polandkolczewski@gmail.com (P.K.);; 2 Department of Functional Anatomy, Pomeranian Medical University in Szczecin, Al. Powstańców Wielkopolskich 72, 70-111 Szczecin, Polandagnieszka.kempinska.podhorodecka@pum.edu.pl (A.K.-P.); 3 Department of Experimental and Clinical Pharmacology, Pomeranian Medical University, Al. Powstańców Wlkp. 72, 70-111 Szczecin, Poland; 4 Department of Gynecologic Oncology and Reconstructive Gynecology, Department of Obstetrics and Gynecology with the High-Risk Pregnancy Unit, Independent Public Specialist Healthcare Centre “Zdroje”, 70-780 Szczecin, Poland; 5 Laboratory of Pharmacodynamics, Pomeranian Medical University in Szczecin, Plac Polskiego Czerwonego Krzyża 1, 71-244 Szczecin, Poland; 6 Department of Gynecology, Endocrinology and Gynecological Oncology, Pomeranian Medical University in Szczecin, Unii Lubelskiej 1, 71-252 Szczecin, Poland

**Keywords:** ovarian cancer, DNA methylation, epigenetics, SEPT9, AKR1B1, biomarker, gene expression, prognosis

## Abstract

Epigenetic dysregulation is increasingly recognized as an important contributor to ovarian carcinogenesis, and may provide novel diagnostic and prognostic biomarkers. This study evaluated DNA methylation and mRNA expression of SEPT9 and AKR1B1 in ovarian cancer and benign ovarian lesions and assessed their clinical significance. Tissue samples were obtained from 27 patients with ovarian cancer and 29 patients with benign ovarian lesions. DNA methylation was analyzed by pyrosequencing and mRNA expression by real-time PCR. Associations with clinicopathological variables, serum CA125 and HE4 concentrations, diagnostic performance, and survival outcomes were evaluated. Receiver operating characteristic (ROC) analysis, multivariable logistic regression, Kaplan–Meier analysis, and Cox regression models were performed. Multiple testing was controlled using the Benjamini–Hochberg false discovery rate procedure. Ovarian cancer tissues demonstrated significantly lower SEPT9 methylation (q = 0.008), higher AKR1B1 methylation (q = 0.0008), and lower AKR1B1 mRNA expression (q = 0.035) compared with benign ovarian lesions. SEPT9 methylation showed the highest diagnostic accuracy (AUC = 0.741). Both SEPT9 methylation (OR = 0.92, *p* = 0.014) and AKR1B1 methylation (OR = 9.69, *p* = 0.008) remained independently associated with ovarian cancer after adjustment for age and menopausal status. Low AKR1B1 mRNA expression was associated with shorter progression-free survival (*p* = 0.0007). These findings indicate that SEPT9 methylation and AKR1B1 dysregulation are associated with ovarian cancer. SEPT9 methylation demonstrated the best discriminative ability among the evaluated biomarkers. Further validation in larger independent cohorts is warranted.

## 1. Introduction

Ovarian cancer remains one of the most lethal gynecological malignancies worldwide and continues to be the leading cause of death among gynecological cancers. The lack of effective screening methods and the absence of characteristic symptoms during the early stages of disease result in diagnosis at advanced clinical stages in the majority of patients. Despite advances in cytoreductive surgery, platinum-based chemotherapy, antiangiogenic treatment, and the introduction of poly (ADP-ribose) polymerase (PARP) inhibitors, long-term survival remains unsatisfactory due to frequent recurrence and treatment resistance. Currently used biomarkers, including cancer antigen 125 (CA125) and human epididymis protein 4 (HE4), are helpful in clinical practice; however, their diagnostic performance remains insufficient, particularly in early-stage disease. Therefore, there is a continuing need to identify novel molecular biomarkers that may improve diagnosis, prognostic assessment, and personalized therapeutic strategies [1,2].

Among epigenetic mechanisms, DNA methylation is one of the most extensively studied. Methylation involves the addition of a methyl group to cytosine residues within CpG dinucleotides. Aberrant methylation patterns, including promoter hypermethylation and global hypomethylation, are frequently observed in malignant tumors. Hypermethylation of promoter-associated CpG islands may suppress gene transcription and contribute to silencing of tumor suppressor genes, whereas hypomethylation may promote genomic instability and activation of oncogenic pathways. Because methylation changes often occur early during carcinogenesis and remain relatively stable, they have emerged as attractive candidates for diagnostic and prognostic biomarker development [3,4].

Recent studies have further emphasized the importance of epigenetic dysregulation in ovarian cancer. Comprehensive analyses of ovarian cancer epigenomes have demonstrated that DNA methylation patterns are associated with tumor subtype, chemoresistance, immune microenvironment characteristics, and patient survival. Moreover, advances in liquid biopsy technologies have highlighted the potential utility of methylation-based biomarkers for early detection, disease monitoring, and prediction of therapeutic response. These findings support the growing role of epigenetic biomarkers in precision oncology and suggest that characterization of novel methylation targets may provide clinically relevant information in ovarian cancer management [3,4,5,6].

Among genes potentially involved in carcinogenesis, SEPT9 has attracted considerable attention. SEPT9 encodes a GTP-binding cytoskeletal protein involved in cytokinesis and cell-cycle regulation. Dysregulation of SEPT9 expression has been described in multiple malignancies, including colorectal, breast, prostate, and ovarian cancers. In colorectal cancer, SEPT9 promoter methylation has become one of the best-validated epigenetic biomarkers and forms the basis of commercially available blood-based diagnostic assays. Furthermore, SEPT9 methylation has been associated with tumor stage, recurrence, and survival outcomes, highlighting its potential clinical utility [7,8,9,10].

Although altered SEPT9 expression has been reported in ovarian tumors, studies investigating SEPT9 methylation in ovarian cancer remain scarce. Scott et al. demonstrated altered transcriptional patterns of SEPT9 during ovarian tumorigenesis [11], whereas Lyu et al. reported elevated plasma SEPT9 levels in patients with serous ovarian cancer [12]. However, the relationship between SEPT9 methylation, gene expression, and clinical outcomes in ovarian cancer remains largely unexplored [11,12].

AKR1B1 encodes an enzyme involved in metabolic and inflammatory pathways as-sociated with carcinogenesis, including NF-κB, cyclooxygenase-mediated signaling, and PI3K/AKT-dependent pathways. Aberrant AKR1B1 expression has been described in colorectal, breast, and endometrial cancers, although its biological role appears to be tumor-specific. In addition, promoter methylation of AKR1B1 has been associated with altered gene expression and malignant transformation in selected tumor types [13,14].

To date, little is known about the biological and clinical significance of AKR1B1 in ovarian cancer. The available evidence is limited mainly to studies demonstrating altered expression of AKR1B1 in ovarian tumor tissue. Moreover, no comprehensive analyses simultaneously evaluating AKR1B1 methylation status and mRNA expression in ovarian cancer have been reported. This represents a substantial gap in current knowledge, particularly considering the increasing interest in AKR1B1 as a potential therapeutic target in oncology [13,15].

Interestingly, a recent methylation panel combining AKR1B1 and SEPT9 achieved a sensitivity of 98% and specificity of 99% for colorectal cancer detection, suggesting that these genes may constitute valuable epigenetic biomarkers and warrant investigation in other malignancies, including ovarian cancer [16]. According to our knowledge, no previous study has simultaneously investigated promoter methylation and gene expression of SEPT9 and AKR1B1 in ovarian cancer. This observation provides the rationale for the present study.

Given the biological functions of SEPT9 and AKR1B1, the evidence linking these genes to carcinogenesis, and the limited available data regarding their epigenetic regulation in ovarian cancer, further investigation appears justified. Simultaneous assessment of promoter methylation and gene expression may provide insight into mechanisms underlying ovarian tumor development and identify novel biomarkers associated with disease progression and prognosis.

We hypothesized that aberrant methylation of SEPT9 and AKR1B1 contributes to altered gene expression in ovarian cancer tissue and that these molecular alterations are associated with clinicopathological characteristics and patient outcomes. Furthermore, we hypothesized that methylation and expression profiles of these genes may differentiate malignant from benign ovarian lesions and therefore possess potential diagnostic and prognostic value.

The aim of the present study was to comprehensively evaluate SEPT9 and AKR1B1 methylation and mRNA expression profiles in ovarian cancer and benign ovarian lesions, investigate their relationships with clinicopathological variables, assess their diagnostic performance, and explore their prognostic significance for progression-free and overall survival. In addition, we examined the relationship between promoter methylation and gene expression to gain further insight into the potential epigenetic regulation of these genes in ovarian cancer.

## 2. Results

### 2.1. Clinical Characteristics of the Study Population

The study included 56 patients, comprising 27 patients with ovarian cancer (R group) and 29 patients with benign ovarian lesions (NR group). Patients with ovarian cancer were significantly older than those in the control group (median age: 66.7 vs. 47.2 years, *p* = 0.002). Consistently, a higher proportion of patients aged ≥65 years was observed in the ovarian cancer group (59.3% vs. 24.1%, *p* = 0.008). Postmenopausal status was also significantly more frequent among ovarian cancer patients (77.8% vs. 41.4%, *p* = 0.006). No significant differences in BMI distribution were observed between groups (*p* = 0.379). The results are presented in Table 1.

### 2.2. Comparison of SEPT9 and AKR1B1 Methylation Levels

Median SEPT9 methylation was significantly lower in ovarian cancer tissues compared with benign ovarian lesions (36.83% [IQR: 22.33–54.67] vs. 53.33% [IQR: 47.67–58.50], *p* = 0.004). Similarly, significant differences were observed for AKR1B1 methylation, with ovarian cancer tissues demonstrating higher methylation levels than benign lesions (2.8% [IQR: 2.4–3.2] vs. 2.2% [IQR: 1.8–2.6], *p* = 0.0002).

To account for multiple testing, *p*-values were adjusted using the Benjamini–Hochberg false discovery rate (FDR) procedure. After FDR correction, significant differences between ovarian cancer and control groups remained for SEPT9 methylation (q = 0.008) and AKR1B1 methylation (q = 0.0008). The results are presented in Table 2.

**Figure 1:**
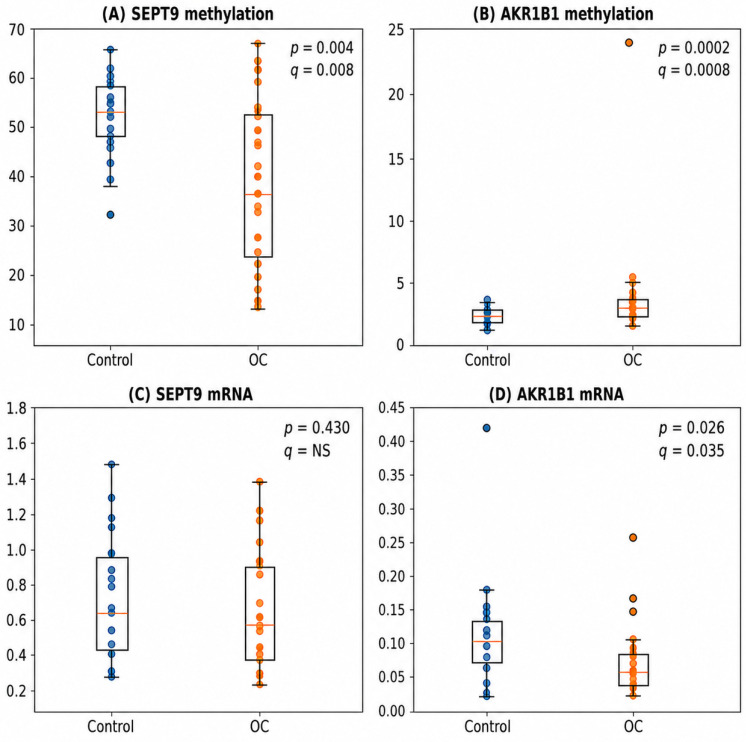
**Distribution of SEPT9 and AKR1B1 methylation levels and mRNA expression in ovarian cancer and control tissues**. Boxplots show median values, interquartile ranges, and individual observations for (**A**) SEPT9 methylation, (**B**) AKR1B1 methylation, (**C**) SEPT9 mRNA expression, and (**D**) AKR1B1 mRNA expression. SEPT9 methylation was significantly lower, whereas AKR1B1 methylation was significantly higher in ovarian cancer tissues compared with benign ovarian lesions. AKR1B1 mRNA expression was significantly reduced in ovarian cancer tissues, while SEPT9 mRNA expression did not differ significantly between groups. Statistical comparisons were performed using the Mann–Whitney U test.

The distribution of SEPT9 and AKR1B1 methylation levels and mRNA expression in ovarian cancer and control tissues is presented in Figure 1.

No significant differences in SEPT9 or AKR1B1 methylation were observed according to FIGO stage, BMI, menopausal status, or histological subtype. The results are presented in Table 3.

CpG-specific methylation analyses for SEPT9 and AKR1B1 are presented in Supplementary Tables S2 and S3, respectively.

### 2.3. Comparison of SEPT9 and AKR1B1 mRNA Expression

SEPT9 mRNA expression did not differ significantly between ovarian cancer and control tissues (0.576 vs. 0.639, *p* = 0.430). Likewise, no significant associations were found between SEPT9 expression and FIGO stage, BMI, menopausal status, or histological subtype.

In contrast, AKR1B1 mRNA expression was significantly lower in ovarian cancer tissue compared with benign ovarian lesions (0.063 [IQR: 0.044–0.082] vs. 0.110 [IQR: 0.072–0.129], *p* = 0.026). No significant differences in AKR1B1 expression were observed according to FIGO stage, BMI, menopausal status, or histological subtype. Following FDR correction, the difference in AKR1B1 mRNA expression remained statistically significant (q = 0.035), whereas SEPT9 mRNA expression remained non-significant. The results are presented in Table 2 and Table 3.

Overall, SEPT9 methylation, AKR1B1 methylation, and AKR1B1 mRNA expression remained significantly different between ovarian cancer and control tissues after correction for multiple testing, supporting the robustness of the observed molecular alterations.

### 2.4. Relationship Between DNA Methylation and mRNA Expression

A significant positive correlation between SEPT9 methylation and SEPT9 mRNA expression was observed in the control group (rho = 0.474, *p* = 0.035). However, this association did not remain statistically significant after correction for multiple testing. Furthermore, this relationship was not present in ovarian cancer tissues (rho = −0.332, *p* = 0.113).

No significant correlations between AKR1B1 methylation and AKR1B1 mRNA expression were identified in either the control group or the ovarian cancer group.

When all samples were analyzed together, neither SEPT9 nor AKR1B1 methylation levels correlated significantly with their corresponding mRNA expression levels.

Detailed correlation analyses are presented in Supplementary Table S1.

### 2.5. Correlation Analyses

Correlation analyses between methylation levels, mRNA expression, and clinical variables are presented in Supplementary Table S1, whereas CpG-specific correlation analyses are provided in Supplementary Tables S4 and S5. Although several nominally significant associations were identified, none remained statistically significant after Benjamini–Hochberg false discovery rate correction.

### 2.6. Receiver Operating Characteristic Analysis

**Figure 2:**
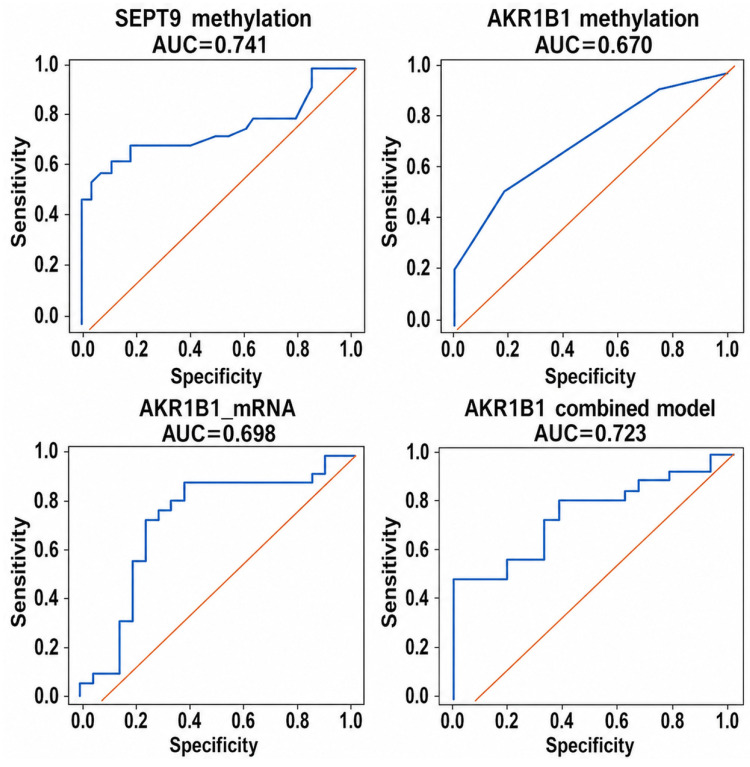
**Receiver operating characteristic (ROC) curves for SEPT9 and AKR1B1 biomarkers in discrimination between ovarian cancer and control tissues.** SEPT9 methylation demonstrated the highest diagnostic performance (AUC = 0.741), followed by the combined AKR1B1 methylation/mRNA model (AUC = 0.723).

Receiver operating characteristic (ROC) analysis demonstrated that SEPT9 methylation achieved the highest diagnostic accuracy among the evaluated biomarkers (AUC = 0.741), followed by the combined AKR1B1 methylation/mRNA model (AUC = 0.723), AKR1B1 mRNA expression (AUC = 0.698), and AKR1B1 methylation (AUC = 0.670). The combined AKR1B1 model achieved 100% specificity at the optimal cut-off value. SEPT9 mRNA expression demonstrated limited diagnostic utility (AUC = 0.571). Detailed ROC parameters are presented in Table 4, and ROC curves are shown in Figure 2.

Among individual biomarkers, SEPT9 methylation demonstrated the highest discriminative ability, whereas integration of AKR1B1 methylation and mRNA expression improved specificity and achieved the highest overall classification performance among AKR1B1-based models.

### 2.7. Multivariable Logistic Regression Analysis

Multivariable logistic regression adjusted for age and menopausal status demonstrated that both AKR1B1 methylation (OR = 9.69, 95% CI: 1.82–51.53, *p* = 0.008) and SEPT9 methylation (OR = 0.92, 95% CI: 0.87–0.98, *p* = 0.014) remained independently associated with ovarian cancer status. In the mRNA-based model, AKR1B1 expression showed borderline significance (*p* = 0.050), whereas SEPT9 expression was not independently associated with ovarian cancer status. Age remained significant only in the mRNA-based model (Table 5).

These findings indicate that the associations of SEPT9 and AKR1B1 methylation with ovarian cancer status were independent of age and menopausal status. Specifically, higher AKR1B1 methylation increased the odds of ovarian cancer nearly tenfold, whereas higher SEPT9 methylation was associated with a lower probability of ovarian cancer, suggesting opposite directions of epigenetic dysregulation for the two genes.

### 2.8. Survival Analyses

**Figure 3:**
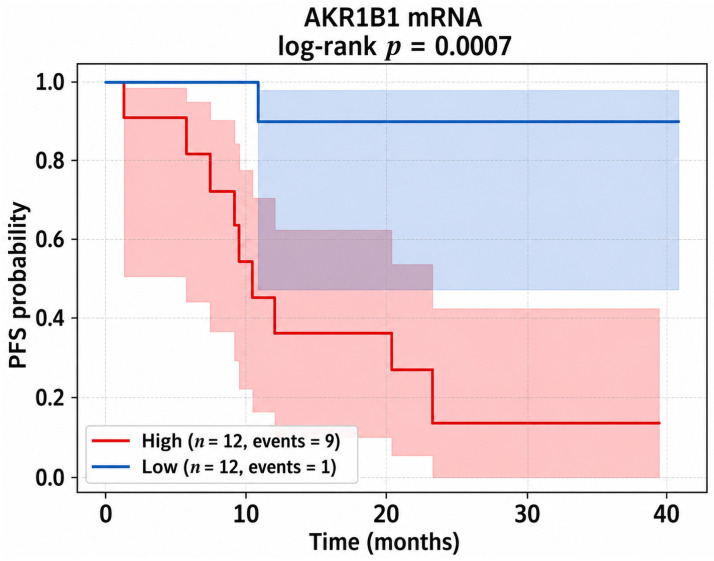
**Kaplan–Meier analysis of progression-free survival according to AKR1B1 mRNA expression.** Patients with high AKR1B1 mRNA expression demonstrated significantly longer progression-free survival than patients with low expression (log-rank *p* = 0.0007).

The median clinical follow-up duration was 28.7 months (IQR 12.3–34.1). During follow-up, 12 patients experienced disease progression and 6 patients died. Kaplan–Meier analysis demonstrated no significant associations between PFS and SEPT9 methylation (*p* = 0.649), SEPT9 mRNA expression (*p* = 0.830), or AKR1B1 methylation (*p* = 0.402). In contrast, patients with low AKR1B1 mRNA expression exhibited significantly shorter progression-free survival than those with high expression (log-rank *p* = 0.0007). Kaplan–Meier curves are presented in Figure 3.

No significant associations were observed between any marker and overall survival.

Additional Kaplan–Meier survival curves are provided in Supplementary Figures S1 and S2.

Consistent with Kaplan–Meier analysis, AKR1B1 mRNA expression was significantly associated with progression-free survival in univariate Cox regression analysis, with patients exhibiting low expression demonstrating poorer outcomes. However, this association did not remain statistically significant in multivariable analysis (HR = 2.192, 95% CI: 0.888–5.409, *p* = 0.0887) (Table 6). No significant associations between SEPT9 methylation, SEPT9 mRNA expression, or AKR1B1 methylation and patient survival outcomes were identified.

Full univariate and multivariable Cox regression results are provided in Supplementary Tables S6–S9.

## 3. Discussion

The present study evaluated DNA methylation and mRNA expression of SEPT9 and AKR1B1 in ovarian cancer tissues and benign ovarian lesions. To our knowledge, this is the first study to simultaneously investigate promoter methylation and gene expression of SEPT9 and AKR1B1 in ovarian cancer. The principal findings of the present study were: (1) significantly lower SEPT9 methylation in ovarian cancer tissues, (2) significantly higher AKR1B1 methylation in ovarian cancer tissues, (3) significantly reduced AKR1B1 mRNA expression in ovarian cancer, and (4) inverse correlations between AKR1B1 methylation and serum CA125 and HE4 concentrations. Collectively, these findings suggest that epigenetic dysregulation of AKR1B1 may contribute to ovarian carcinogenesis and possess potential clinical relevance.

Importantly, the observed differences in SEPT9 methylation, AKR1B1 methylation, and AKR1B1 mRNA expression remained statistically significant after correction for multiple testing using the Benjamini–Hochberg false discovery rate procedure.

Epigenetic alterations are increasingly recognized as critical drivers of ovarian cancer development and progression. DNA methylation represents one of the most extensively investigated epigenetic mechanisms and may contribute to malignant transformation through transcriptional silencing of genes involved in cell-cycle control, apoptosis, DNA repair, and cellular differentiation. Recent studies have highlighted the growing importance of methylation-based biomarkers in ovarian cancer diagnosis, prognosis, and treatment stratification [3,5,6,17]. These observations support ongoing efforts to identify novel epigenetic markers involved in ovarian tumor biology.

SEPT9 has attracted considerable attention because of its established role as a methylation biomarker in colorectal cancer. Aberrant SEPT9 methylation has been associated with tumor development, recurrence, and survival outcomes, and circulating methylated SEPT9 DNA is currently used in clinically available blood-based screening assays [18,19]. However, information regarding the role of SEPT9 in ovarian cancer remains limited. Scott et al. demonstrated altered SEPT9 transcription patterns during ovarian tumorigenesis, suggesting that dysregulation of SEPT9 may occur during the development of ovarian malignancies [11].

In the present study SEPT9 methylation was significantly lower in ovarian cancer tissues than in benign ovarian lesions. This observation differs from findings reported for colorectal cancer, where SEPT9 hypermethylation is commonly observed [9,18,19]. Such discrepancies may reflect tissue-specific functions of SEPT9 and the molecular heterogeneity of ovarian cancer, including diverse molecular pathways and histological subtypes.

Despite the observed methylation differences, SEPT9 mRNA expression did not differ significantly between ovarian cancer and control tissues. Moreover, no associations were identified between SEPT9 methylation and clinicopathological variables. These findings suggest that methylation of the analyzed SEPT9 region may not represent the dominant mechanism regulating SEPT9 transcription in ovarian cancer. Alternative mechanisms, including histone modifications, chromatin remodeling, transcription factor activity, or post-transcriptional regulation, may play a more important role. Interestingly, a significant positive correlation between SEPT9 methylation and mRNA expression was observed only in the control group, whereas this relationship disappeared in ovarian cancer tissues, potentially indicating disruption of normal epigenetic regulation during malignant transformation.

The most noteworthy findings of the present study concern AKR1B1. We observed significantly increased AKR1B1 methylation accompanied by significantly reduced AKR1B1 mRNA expression in ovarian cancer tissues. This pattern is consistent with a potential epigenetic silencing mechanism, in which promoter hypermethylation contributes to transcriptional repression. Although a direct correlation between methylation and expression was not demonstrated, the simultaneous occurrence of hypermethylation and reduced mRNA levels supports the hypothesis that epigenetic mechanisms may contribute to AKR1B1 downregulation in ovarian cancer. The absence of a significant correlation may reflect the multifactorial regulation of AKR1B1 expression, which is likely influenced by additional epigenetic, transcriptional, and post-transcriptional mechanisms.

AKR1B1 encodes a member of the aldo-keto reductase family and participates in glucose metabolism, prostaglandin synthesis, oxidative stress responses, and inflammatory signaling pathways. Previous studies have demonstrated interactions between AKR1B1 and several pathways involved in carcinogenesis, including NF-κB and PI3K/AKT signaling [13,20]. Consequently, AKR1B1 has been proposed as a potential regulator of tumor progression and a possible therapeutic target in several malignancies.

Data regarding AKR1B1 in ovarian cancer remain scarce. Hojnik et al. evaluated AKR1B1 protein expression in high-grade serous ovarian carcinoma and demonstrated that higher AKR1B1 expression was associated with improved disease-free and overall survival [15]. Moreover, AKR1B1 remained an independent prognostic factor in multivariable analyses, suggesting a potentially protective role in ovarian cancer biology.

Our findings are partially consistent with these observations, as reduced AKR1B1 mRNA expression and increased promoter methylation may indicate epigenetic-mediated downregulation during ovarian tumorigenesis. Direct comparisons with existing literature are difficult because data regarding AKR1B1 methylation in ovarian cancer remain extremely limited [13,14,15]. The combined observation of increased AKR1B1 promoter methylation, decreased mRNA expression, and poorer progression-free survival among patients with lower AKR1B1 expression provides indirect evidence that epigenetic dysregulation of AKR1B1 may contribute to ovarian cancer progression.

Although methylation markers were not associated with patient outcomes, low AKR1B1 mRNA expression was significantly associated with shorter progression-free survival in Kaplan–Meier and univariate Cox analyses. This observation supports a potential protective role of AKR1B1 in ovarian cancer biology and is consistent with previous findings reported by Hojnik et al. [15]. Although statistical significance was not retained in multivariable Cox models, the observed effect size remained substantial, suggesting a potential prognostic role of AKR1B1 in ovarian cancer. Given the limited cohort size and number of progression events, these findings should be interpreted cautiously and require validation in larger independent cohorts.

An additional finding of potential clinical importance was the inverse association between AKR1B1 methylation and serum CA125 and HE4 concentrations. Although these findings should be interpreted cautiously, they may indicate a relationship between AKR1B1 methylation and biological processes associated with disease burden. Given the widespread clinical use of CA125 and HE4 in ovarian cancer diagnosis and monitoring, further investigation of the relationship between AKR1B1 methylation and established biomarkers appears warranted.

SEPT9 methylation demonstrated the highest diagnostic accuracy among the evaluated biomarkers (AUC = 0.741), whereas AKR1B1 methylation and AKR1B1 mRNA expression showed moderate diagnostic performance. The combined AKR1B1 methylation/mRNA model further improved specificity, supporting the complementary diagnostic value of epigenetic and transcriptional alterations. Although the diagnostic performance of AKR1B1 methylation alone was moderate (AUC = 0.670), its independent association with ovarian cancer status in multivariable logistic regression highlights its potential biological and diagnostic relevance. This observation is particularly interesting in light of previous studies demonstrating promising diagnostic performance of methylation-based panels containing AKR1B1 and SEPT9 in other malignancies [16]. Therefore, AKR1B1 methylation may represent a candidate biomarker for future multimarker diagnostic models integrating molecular and clinical variables. Future multimarker diagnostic models may benefit from integrating epigenetic biomarkers with complementary molecular, metabolic, and clinical markers. Recent studies have highlighted the contribution of transferrin-related mechanisms to ovarian carcinogenesis and suggested the potential utility of plasma transferrin as a biomarker, supporting the concept that combining biomarkers reflecting distinct biological pathways may further improve diagnostic performance [21,22].

Importantly, both SEPT9 methylation and AKR1B1 methylation remained independently associated with ovarian cancer status after adjustment for age and menopausal status in multivariable logistic regression models. Because patients with ovarian cancer were significantly older and more frequently postmenopausal than controls, residual confounding cannot be completely excluded despite adjustment for age and menopausal status in multivariable analyses. Furthermore, given that DNA methylation patterns may undergo age-related changes, residual confounding associated with biological aging cannot be completely excluded despite statistical adjustment. Future studies involving larger age-matched cohorts are warranted to further validate these findings. The independent association of both methylation markers with ovarian cancer status strengthens the biological relevance of the observed methylation alterations and suggests that these markers may provide diagnostic information beyond conventional demographic risk factors.

Notably, neither methylation levels nor mRNA expression differed significantly according to FIGO stage, histological subtype, BMI, or menopausal status. These findings may suggest that the observed molecular alterations are not strongly associated with tumor stage or the other evaluated clinicopathological characteristics. However, the limited sample size may have reduced the statistical power required to detect weaker associations.

The strengths of the present study include the simultaneous evaluation of DNA methylation and mRNA expression, the inclusion of a control group consisting of benign ovarian lesions, and the assessment of relationships with clinically relevant biomarkers. To our knowledge, this is the first study to simultaneously evaluate SEPT9 and AKR1B1 promoter methylation together with mRNA expression and survival outcomes in ovarian cancer [12,15]. While previous studies primarily focused on gene expression or protein levels, investigations integrating methylation and transcriptional activity remain scarce. Several limitations should also be acknowledged. First, the study was conducted in a relatively small cohort from a single institution. Second, protein expression was not assessed, preventing confirmation that the observed molecular alterations translated into changes at the protein level. Third, the cross-sectional design precludes direct assessment of causality between methylation changes and altered gene expression. Although survival analyses were performed, the limited number of progression and death events reduced statistical power and may have affected the stability of multivariable prognostic models. Several regression estimates demonstrated wide confidence intervals, reflecting the limited sample size and number of outcome events. Therefore, the multivariable regression models should be interpreted as exploratory and hypothesis-generating rather than confirmatory. Another important limitation of the present study is the lack of an independent external validation cohort. Although publicly available resources such as TCGA and GEO were considered, we did not identify a dataset directly comparable to our study design, particularly with respect to the inclusion of benign ovarian lesions and matched methylation and gene expression data for both SEPT9 and AKR1B1. Validation in larger, multicenter independent cohorts and functional studies investigating the mechanistic relationship between AKR1B1 methylation and transcriptional regulation is therefore warranted.

A limitation of the present study is its observational design, which precludes drawing conclusions regarding the causal role of AKR1B1 hypermethylation in ovarian cancer progression. Although significant associations were identified between AKR1B1 methylation, gene expression, and clinicopathological features, these findings should not be interpreted as evidence of a direct mechanistic relationship. Further functional studies, including in vitro and in vivo experiments, are required to determine whether AKR1B1 hypermethylation directly contributes to ovarian cancer progression and to elucidate the underlying molecular mechanisms. Additionally, protein-level validation would provide further insight into the biological significance of the observed transcriptional changes. Although the investigated biomarkers demonstrated high diagnostic accuracy, as reflected by the obtained area under the receiver operating characteristic curve (AUC) values, their sensitivity and specificity may limit their use as standalone diagnostic markers in clinical practice. Future studies should also evaluate the potential improvement of diagnostic performance through the integration of biomarker panels combining molecular and clinical parameters. Another limitation of the present study is the heterogeneity of the ovarian cancer cohort, which included different histological subtypes. Although this approach reflects the biological complexity of ovarian cancer observed in clinical settings, it may also contribute to variability in molecular profiles. Furthermore, subgroup analyses were limited by the available sample size and should therefore be interpreted cautiously. Future studies with larger, subtype-specific cohorts are needed to validate these findings and determine the potential differences in biomarker patterns among distinct ovarian cancer subtypes.

## 4. Materials and Methods

### 4.1. Study Population

The present study addresses molecular targets, including DNA methylation and mRNA expression of SEPT9 and AKR1B1. The study was conducted in accordance with the Declaration of Helsinki and approved by the Bioethics Committee of the Pomeranian Medical University in Szczecin (approval no. KB-0012/55/2021, 20 December 2021). Written informed consent was obtained from all participants prior to enrollment.

This retrospective observational study included 56 women who underwent surgery for an ovarian lesion or tumor. The study cohort consisted of 27 patients with histopathologically confirmed ovarian cancer and 29 patients with benign ovarian lesions serving as the control group.

Clinical and demographic data were obtained from medical records and included age, body mass index (BMI), menopausal status, serum CA125 concentration, serum HE4 concentration, histological subtype, and FIGO stage. Histopathological classification was established according to the World Health Organization (WHO) criteria, while tumor stage was determined according to the International Federation of Gynecology and Obstetrics (FIGO) classification.

### 4.2. Tissue Collection

Tumor tissue samples were obtained during surgery from patients with ovarian cancer. Control samples were collected from women undergoing surgical treatment for benign ovarian lesions. Following excision, tissue specimens were immediately secured and stored under appropriate conditions until molecular analyses were performed.

### 4.3. DNA Isolation and Methylation Analysis

Total DNA was isolated from approximately 25 mg of each tissue sample using the GeneMATRIX Tissue DNA Purification Kit (EURX, Gdańsk, Poland), according to the manufacturer’s instructions.

Bisulfite conversion was performed using the EpiTect Fast DNA Bisulfite Kit (Qiagen, Hilden, Germany). Converted DNA served as a template for polymerase chain reaction (PCR) amplification of selected promoter regions containing CpG sites within the *SEPT9* and *AKR1B1* genes.

Quantitative methylation analysis was performed using pyrosequencing on a PyroMark Q48 Autoprep instrument (Qiagen, Hilden, Germany) with PyroMark Q48 Advanced CpG Reagents and commercially available PyroMark CpG assays (Hs_SEPT9_01_PM00182903 and Hs_AKR1B1_01_PM00125622). Human Methylated and Non-Methylated DNA Set (Zymo Research, Irvine, CA, USA) was used as positive and negative methylation controls.

Methylation levels were expressed as percentages of methylated cytosines within analyzed CpG sites. For statistical analyses, the mean methylation level of all analyzed CpG sites within a given gene was used.

### 4.4. RNA Isolation and Quantitative Real-Time PCR

Total RNA was isolated from approximately 20–30 mg of tissue using the Direct-zol™ RNA MiniPrep Plus Kit (Zymo Research, Irvine, CA, USA). RNA concentration and purity were evaluated spectrophotometrically using a DeNovix DS-11 FX+ spectrophotometer (DeNovix Inc., Wilmington, DE, USA).

Complementary DNA (cDNA) synthesis was performed using the SuperScript™ IV VILO™ Reverse Transcription Kit (Invitrogen™, Waltham, MA, USA) according to the manufacturer’s protocol.

Quantitative real-time PCR (qRT-PCR) was conducted using a ViiA™ 7 Real-Time PCR System (Applied Biosystems, Waltham, MA, USA) and predesigned TaqMan Gene Expression Assays for SEPT9 and AKR1B1. Each sample was analyzed in duplicate, and mean cycle threshold (Ct) values were used for subsequent calculations.

Relative gene expression levels were determined using the 2^−ΔCt^ method. ACTB, GAPDH, GUSB, PPIA, and RPLP0 served as endogenous reference genes.

### 4.5. Statistical Analysis

Statistical analyses were performed using Statistica 13.3 software (TIBCO Software Inc., Palo Alto, CA, USA) and R statistical software version 4.3.2 (R Foundation for Statistical Computing, Vienna, Austria).

Continuous variables were expressed as median and interquartile range (IQR), while categorical variables were presented as numbers and percentages.

Comparisons between two independent groups were performed using the Mann–Whitney U test. Associations between categorical variables were evaluated using the χ^2^ test.

Correlations between methylation levels, mRNA expression, and clinical variables were assessed using Spearman’s rank correlation coefficient (rho).

To account for multiple testing, *p*-values were adjusted using the Benjamini–Hochberg false discovery rate (FDR) procedure. FDR-adjusted q-values < 0.05 were considered statistically significant.

CpG-specific methylation analyses and detailed correlation analyses are presented in the Supplementary Materials.

The diagnostic performance of SEPT9 and AKR1B1 methylation levels and mRNA expression was evaluated using receiver operating characteristic (ROC) curve analysis. The area under the curve (AUC), sensitivity, specificity, and optimal cut-off values based on the Youden index were calculated.

Multivariable logistic regression analyses were performed to evaluate independent associations between methylation levels or mRNA expression and ovarian cancer status. Models were adjusted for age and menopausal status. Odds ratios (ORs) with 95% confidence intervals (CIs) were calculated.

Progression-free survival (PFS) was defined as the time from primary treatment to disease progression or recurrence. Overall survival (OS) was defined as the time from diagnosis to death from any cause or last follow-up. Disease progression and recurrence were defined according to RECIST criteria and confirmed by imaging and/or clinical evaluation.

Survival curves were estimated using the Kaplan–Meier method and compared using the log-rank test. Univariate and multivariate Cox proportional hazards regression models were used to identify independent prognostic factors for PFS and OS.

For survival analyses, molecular markers were evaluated both as continuous variables (per 1 standard deviation increase) and as dichotomized variables (high versus low expression/methylation based on median values). Variables with *p* < 0.10 in univariate Cox regression analyses were considered candidates for multivariable models. Multivariable Cox models were adjusted for FIGO stage, histological subtype, and surgical approach (PDS vs. IDS), depending on endpoint and variable availability.

All statistical tests were two-sided, and *p*-values < 0.05 were considered statistically significant.

## 5. Conclusions

Ovarian cancer tissues exhibited reduced SEPT9 methylation, increased AKR1B1 methylation, and decreased AKR1B1 mRNA expression compared with benign ovarian lesions. SEPT9 methylation demonstrated the highest diagnostic performance among the evaluated biomarkers and remained independently associated with ovarian cancer status after adjustment for age and menopausal status. Low AKR1B1 mRNA expression was associated with shorter progression-free survival, suggesting a possible association between AKR1B1 expression and disease progression in ovarian cancer. These findings suggest that SEPT9 methylation may have diagnostic utility, whereas AKR1B1 may represent a biologically relevant component of ovarian carcinogenesis. The association between AKR1B1 expression and progression-free survival suggests its potential clinical relevance. Further validation in larger, multicenter independent cohorts is warranted.

## Figures and Tables

**Table 1:** Clinicodemographic and clinicopathological characteristics of the study population.

Variable	Total Cohort (*n* = 56)	Ovarian Cancer (*n* = 27)	Control Group (*n* = 29)	*p*-Value
**Age (years), median (IQR)**	58.2 (47.1–70.4)	66.7 (54.1–71.4)	47.2 (35.3–63.1)	0.002
**BMI (kg/m^2^), median (IQR)**	26.3 (23.2–31.0)	26.5 (24.0–31.1)	25.7 (22.5–30.8)	0.486
**Age < 65 years, *n* (%)**	33 (58.9)	11 (40.7)	22 (75.9)	0.008
**Age ≥ 65 years, *n* (%)**	23 (41.1)	16 (59.3)	7 (24.1)	
**BMI < 25 kg/m^2^, *n* (%)**	22 (39.3)	9 (33.3)	13 (44.8)	0.379
**BMI ≥ 25 kg/m^2^, *n* (%)**	34 (60.7)	18 (66.7)	16 (55.2)	
**Premenopausal, *n* (%)**	23 (41.1)	6 (22.2)	17 (58.6)	0.006
**Postmenopausal, *n* (%)**	33 (58.9)	21 (77.8)	12 (41.4)	
**Clinicopathological characteristics of ovarian cancer patients**				
**Variable**		*n* (%)		
**FIGO I–II**		8 (29.6)		
**FIGO III–IV**		19 (70.4)		
**High-grade serous carcinoma**		19 (70.4)		
Other histological types *		8 (29.6)		

Abbreviations: BMI, body mass index; FIGO, International Federation of Gynecology and Obstetrics; IQR, interquartile range. * Other histological types included clear cell, endometrioid, low-grade serous, and mucinous ovarian carcinomas.

**Table 2:** Comparison of SEPT9 and AKR1B1 methylation levels and mRNA expression between ovarian cancer and control groups.

Marker	Ovarian Cancer Median (IQR)	Control Group Median (IQR)	*p*-Value	q-Value (FDR)
**SEPT9 methylation (%)**	36.83 (22.33–54.67)	53.33 (47.67–58.50)	0.004	0.008
**AKR1B1 methylation (%)**	2.8 (2.4–3.2)	2.2 (1.8–2.6)	0.0002	0.0008
**SEPT9 mRNA expression**	0.576 (0.365–0.862)	0.639 (0.428–0.898)	0.430	NS
**AKR1B1 mRNA expression**	0.063 (0.044–0.082)	0.110 (0.072–0.129)	0.026	0.035

Abbreviations: FDR, false discovery rate; IQR, interquartile range.

**Table 3:** Associations of SEPT9 and AKR1B1 methylation levels and mRNA expression with clinicopathological variables.

Variable	SEPT9 Methylation (%) Median (IQR)	*p*-Value	AKR1B1 Methylation (%) Median (IQR)	*p*-Value	SEPT9 mRNA Median (IQR)	*p*-Value	AKR1B1 mRNA Median (IQR)	*p*-Value
**FIGO I–II**	41.75 (25.44–51.25)	1.000	2.7 (2.4–3.8)	0.810	0.556 (0.380–0.877)	0.976	0.045 (0.036–0.072)	0.134
**FIGO III–IV**	36.83 (22.33–61.17)		2.8 (2.4–3.2)		0.592 (0.365–0.838)		0.069 (0.055–0.089)	
**High-grade serous carcinoma**	40.17 (22.33–61.17)	0.541	2.6 (2.2–3.2)	0.092	0.592 (0.322–0.862)	0.830	0.069 (0.047–0.082)	0.646
**Other histological types**	36.75 (19.83–50.75)		3.2 (2.7–3.8)		0.556 (0.430–0.853)		0.058 (0.039–0.110)	
**BMI ≤ 25 kg/m^2^**	48.25 (36.83–58.50)	0.867	2.3 (1.8–2.8)	0.473	0.628 (0.320–0.829)	0.625	0.081 (0.056–0.121)	0.858
**BMI > 25 kg/m^2^**	49.33 (36.67–55.83)		2.4 (2.2–2.8)		0.592 (0.424–0.888)		0.074 (0.045–0.117)	
**Premenopausal**	52.00 (37.67–55.17)	0.894	2.2 (2.0–2.8)	0.188	0.476 (0.338–0.829)	0.399	0.079 (0.056–0.122)	0.630
**Postmenopausal**	47.67 (30.83–59.00)		2.4 (2.2–2.8)		0.623 (0.460–0.888)		0.075 (0.045–0.104)	

After Benjamini–Hochberg FDR correction, none of the associations with clinicopathological variables remained statistically significant. Abbreviations: BMI, body mass index; FIGO, International Federation of Gynecology and Obstetrics; IQR, interquartile range.

**Table 4:** Diagnostic performance of SEPT9 and AKR1B1 biomarkers.

Marker	AUC	95% CI	ROC Direction	Cut-Off	Sensitivity (%)	Specificity (%)
**SEPT9 methylation**	0.741	0.610–0.872	Reversed	39.0	66.7	82.8
**SEPT9 mRNA**	0.571	0.420–0.722	Reversed	0.602	62.5	60.0
**AKR1B1 methylation**	0.670	0.528–0.812	Standard	3.0	48.1	79.3
**AKR1B1 mRNA**	0.698	0.560–0.836	Reversed	0.100	87.5	60.0
**AKR1B1 methylation + mRNA model**	0.723	0.589–0.857	Standard	0.640	45.8	100.0

For SEPT9 methylation, SEPT9 mRNA, and AKR1B1 mRNA, ROC direction was reversed because lower marker values were associated with ovarian cancer status. Abbreviations: AUC, area under the curve; ROC, receiver operating characteristic.

**Table 5:** Multivariable logistic regression models for ovarian cancer status. (**A**) Methylation model (*n* = 56); (**B**) mRNA model (*n* = 44).

(**A**)		
**Variable**	**OR (95% CI)**	***p*** **-Value**
**AKR1B1 methylation**	9.69 (1.82–51.53)	0.008
**SEPT9 methylation**	0.92 (0.87–0.98)	0.014
**Age**	1.01 (0.93–1.10)	0.789
**Menopausal status**	6.18 (0.38–101.41)	0.202
(**B**)		
**Variable**	**OR (95% CI)**	***p*** **-Value**
**AKR1B1 mRNA**	<0.001 (≈0–1.00)	0.050
**SEPT9 mRNA**	0.07 (0.004–1.34)	0.078
**Age**	1.16 (1.03–1.30)	0.013
**Menopausal status**	0.28 (0.02–4.66)	0.376

Abbreviations: CI, confidence interval; OR, odds ratio.

**Table 6:** Cox proportional hazards regression analyses for progression-free survival and overall survival.

Endpoint	Analysis	Variable	*n*	Events	HR	95% CI	*p*-Value
**PFS**	Univariate	FIGO III–IV vs. I–II	27	12	4.202	0.866–20.380	0.0748
**PFS**	Univariate	High-grade serous carcinoma vs. other	27	12	8.923	1.127–70.659	0.0382
**PFS**	Univariate	IDS vs. PDS	27	12	3.076	0.994–9.516	0.0512
**PFS**	Univariate	AKR1B1 mRNA (per 1 SD)	24	10	1.623	0.811–3.247	0.1710
**PFS**	Univariate	AKR1B1 mRNA (high vs. low)	27	12	4.614	1.425–14.937	0.0107
**PFS**	Multivariable	AKR1B1 mRNA (high vs. low)	27	12	2.192	0.888–5.409	0.0887
**OS**	Univariate	IDS vs. PDS	27	6	3.856	0.839–17.721	0.0829
**OS**	Univariate	AKR1B1 mRNA (per 1 SD)	24	4	1.422	0.497–4.071	0.5119
**OS**	Univariate	AKR1B1 mRNA (high vs. low)	27	6	1.415	0.330–6.069	0.6400
**OS**	Multivariable	AKR1B1 mRNA (high vs. low)	27	6	1.073	0.441–2.606	0.8771

Only clinically relevant variables and molecular markers demonstrating the strongest associations are presented. Full univariate and multivariable Cox regression results are provided in Supplementary Tables S6–S9. Multivariable models were adjusted for relevant clinicopathological covariates. Abbreviations: CI, confidence interval; HR, hazard ratio; IDS, interval debulking surgery; OS, overall survival; PDS, primary debulking surgery; PFS, progression-free survival; SD, standard deviation.

## Data Availability

The data supporting the findings of this study are available on request from the corresponding author.

## Supplementary Materials

The following supporting information can be downloaded at: https://www.mdpi.com/article/10.3390/ijms27146336/s1.
