# Supplementary material for: DNA Methylation and mRNA Expression of SEPT9 and AKR1B1 in Ovarian Cancer: Diagnostic and Prognostic Implications
Source: Int J Mol Sci. 2026 Jul 16;27(14):6336. doi: 10.3390/ijms27146336 (PMC13410329; doi:10.3390/ijms27146336)
Supplement: Supplementary file 1 [file ijms-27-06336-s001.zip › ijms-4420217-supplementary.pdf]

**Supplementary Table S1**

**Supplementary Table S1. Correlations between methylation levels, mRNA expression, and clinical variables in ovarian cancer and control groups.**

| Variables | rho | p-value |
|---|---|---|
| SEPT9 methylation vs SEPT9 mRNA (control group) | 0.474 | 0.035 |
| SEPT9 methylation vs SEPT9 mRNA (ovarian cancer) | -0.332 | 0.113 |
| AKR1B1 methylation vs AKR1B1 mRNA (control group) | -0.042 | 0.967 |
| AKR1B1 methylation vs AKR1B1 mRNA (ovarian cancer) | -0.302 | 0.765 |
| AKR1B1 methylation vs CA125 | -0.421 | 0.036 |
| AKR1B1 methylation vs HE4 | -0.405 | 0.049 |

*Spearman rank correlation analysis was performed to evaluate associations between methylation levels, mRNA expression, age, BMI, CA125, and HE4 concentrations. None of the correlations remained statistically significant after Benjamini–Hochberg false discovery rate correction.*
***Abbreviations:*** *BMI, body mass index; CA125, cancer antigen 125; HE4, human epididymis protein 4.*

**Supplementary Table S2**

**Supplementary Table S2. CpG-specific methylation analysis of SEPT9 in ovarian cancer and control tissues.**

| CpG site | Ovarian cancer Median (%) | Control Median (%) | p-value | q-value (BH-FDR) |
|---|---|---|---|---|
| **CpG1** | 30 | 46 | 0.0020 | 0.0040 |
| **CpG2** | 30 | 43 | 0.0075 | 0.0090 |
| **CpG3** | 46 | 66 | 0.0026 | 0.0040 |
| **CpG4** | 41 | 59 | 0.0027 | 0.0040 |
| **CpG5** | 48 | 61 | 0.0155 | 0.0155 |
| **CpG6** | 33 | 45 | 0.0013 | 0.0040 |

*Methylation percentages of individual SEPT9 CpG sites were compared between ovarian cancer and benign ovarian lesion tissues using the Mann–Whitney U test. False discovery rate correction was performed using the Benjamini–Hochberg procedure.*
***Abbreviations:*** *CpG, cytosine-phosphate-guanine dinucleotide; FDR, false discovery rate; IQR, interquartile range.*

**Supplementary Table S3**

**Supplementary Table S3. CpG-specific methylation analysis of AKR1B1 in ovarian cancer and control tissues.**

| CpG site | Ovarian cancer Median (%) | Control Median (%) | p-value | q-value (BH-FDR) |
|---|---|---|---|---|
| **CpG1** | 2 | 2 | 0.0199 | 0.0249 |
| **CpG2** | 3 | 2 | 0.0077 | 0.0198 |
| **CpG3** | 2 | 2 | 0.0030 | 0.0150 |
| **CpG4** | 3 | 3 | 0.0119 | 0.0198 |
| **CpG5** | 3 | 3 | 0.0498 | 0.0498 |

*Methylation percentages of individual AKR1B1 CpG sites were compared between ovarian cancer and benign ovarian lesion tissues using the Mann–Whitney U test. False discovery rate correction was performed using the Benjamini–Hochberg procedure.*
***Abbreviations:*** *CpG, cytosine-phosphate-guanine dinucleotide; FDR, false discovery rate; IQR, interquartile range.*

**Supplementary Table S4**

**Supplementary Table S4. Associations between SEPT9 CpG methylation levels and SEPT9 mRNA expression.**

| CpG site | Spearman rho | p-value | q-value (BH-FDR) |
|---|---|---|---|
| **CpG1** | -0.006 | 0.968 | 0.968 |
| **CpG2** | -0.144 | 0.351 | 0.922 |
| **CpG3** | 0.055 | 0.722 | 0.922 |
| **CpG4** | 0.082 | 0.596 | 0.922 |
| **CpG5** | 0.063 | 0.683 | 0.922 |
| **CpG6** | 0.046 | 0.768 | 0.922 |

*Spearman rank correlation analysis was used to assess the relationship between methylation levels of individual SEPT9 CpG sites and SEPT9 mRNA expression levels.*
***Abbreviations:*** *CpG, cytosine-phosphate-guanine dinucleotide.*

## Supplementary Table S5

## Supplementary Table S5. Associations between AKR1B1 CpG methylation levels and AKR1B1 mRNA expression.

| CpG site | Spearman rho | p-value | q-value (BH-FDR) |
|---|---|---|---|
| **CpG1** | -0.119 | 0.441 | 0.635 |
| **CpG2** | -0.138 | 0.373 | 0.635 |
| **CpG3** | -0.102 | 0.508 | 0.635 |
| **CpG4** | -0.095 | 0.540 | 0.635 |
| **CpG5** | -0.199 | 0.196 | 0.635 |

*Spearman rank correlation analysis was used to assess the relationship between methylation levels of individual AKR1B1 CpG sites and AKR1B1 mRNA expression levels.*
***Abbreviations:*** *CpG, cytosine-phosphate-guanine dinucleotide.*

## Supplementary Table S6

## Univariate Cox proportional hazards regression analyses for progression-free survival (PFS)

| Variable | n | Events | HR | 95% CI | p-value |
|---|---|---|---|---|---|
| **Age (per year)** | 27 | 12 | 1.041 | 0.986–1.099 | 0.1461 |
| **FIGO III–IV vs I–II** | 27 | 12 | 4.202 | 0.866–20.380 | 0.0748 |
| **HGSC vs Other** | 27 | 12 | 8.923 | 1.127–70.659 | 0.0382 |
| **IDS vs PDS** | 27 | 12 | 3.076 | 0.994–9.516 | 0.0512 |
| **SEPT9 methylation (mean CpG) per 1 SD** | 27 | 12 | 0.855 | 0.473–1.546 | 0.6051 |
| **SEPT9 methylation High vs Low** | 27 | 12 | 0.790 | 0.269–2.320 | 0.6678 |
| **AKR1B1 methylation (mean CpG) per 1 SD** | 27 | 12 | 0.780 | 0.215–2.832 | 0.7053 |
| **AKR1B1 methylation High vs Low** | 27 | 12 | 0.647 | 0.219–1.910 | 0.4307 |
| **SEPT9 mRNA per 1 SD** | 24 | 10 | 0.903 | 0.501–1.625 | 0.7328 |
| **SEPT9 mRNA High vs Low** | 27 | 12 | 0.992 | 0.338–2.910 | 0.9888 |
| **AKR1B1 mRNA per 1 SD** | 24 | 10 | 1.623 | 0.811–3.247 | 0.1710 |
| **AKR1B1 mRNA High vs Low** | 27 | 12 | 4.614 | 1.425–14.937 | 0.0107 |

Univariate Cox proportional hazards regression analyses were performed to evaluate associations between clinicopathological variables, methylation levels, mRNA expression, and patient outcomes.
**Abbreviations:** CI, confidence interval; HGSC, high-grade serous carcinoma; HR, hazard ratio; IDS, interval debulking surgery; PDS, primary debulking surgery; SD, standard deviation.

## Supplementary Table S7

## Supplementary Table S7. Univariate Cox proportional hazards regression analyses for overall survival (OS)

| Variable | n | Events | HR | 95% CI | p-value |
|---|---|---|---|---|---|
| **Age (per year)** | 27 | 6 | 1.055 | 0.976–1.140 | 0.1812 |
| **FIGO III–IV vs I–II** | 27 | 6 | 5.489 | 0.570–52.892 | 0.1407 |
| **HGSC vs Other** | 27 | 6 | 4.957 | 0.495–49.670 | 0.1734 |
| **IDS vs PDS** | 27 | 6 | 3.856 | 0.839–17.721 | 0.0829 |
| **SEPT9 methylation (mean CpG) per 1 SD** | 27 | 6 | 1.114 | 0.510–2.435 | 0.7861 |
| **SEPT9 methylation High vs Low** | 27 | 6 | 1.410 | 0.312–6.370 | 0.6554 |
| **AKR1B1 methylation (mean CpG) per 1 SD** | 27 | 6 | 0.768 | 0.196–3.008 | 0.7050 |
| **AKR1B1 methylation High vs Low** | 27 | 6 | 0.927 | 0.219–3.931 | 0.9184 |
| **SEPT9 mRNA per 1 SD** | 24 | 4 | 0.736 | 0.286–1.893 | 0.5241 |
| **SEPT9 mRNA High vs Low** | 27 | 6 | 0.629 | 0.140–2.816 | 0.5441 |
| **AKR1B1 mRNA per 1 SD** | 24 | 4 | 1.422 | 0.497–4.071 | 0.5119 |
| **AKR1B1 mRNA High vs Low** | 27 | 6 | 1.415 | 0.330–6.069 | 0.6400 |

*Univariate Cox proportional hazards regression analyses were performed to evaluate associations between clinicopathological variables, methylation levels, mRNA expression, and overall survival.*
***Abbreviations:*** *CI, confidence interval; HGSC, high-grade serous carcinoma; HR, hazard ratio; IDS, interval debulking surgery; OS, overall survival; PDS, primary debulking surgery; SD, standard deviation.*

## Supplementary Table S8. Multivariable Cox proportional hazards regression analyses for progression-free survival (PFS)

| Marker model | n | Events | HR | 95% CI | p-value |
|---|---|---|---|---|---|
| **SEPT9 methylation (mean CpG) per 1 SD** | 27 | 12 | 0.933 | 0.576–1.510 | 0.7768 |
| **SEPT9 methylation High vs Low** | 27 | 12 | 0.944 | 0.383–2.329 | 0.9009 |
| **AKR1B1 methylation (mean CpG) per 1 SD** | 27 | 12 | 0.918 | 0.502–1.678 | 0.7805 |
| **AKR1B1 methylation High vs Low** | 27 | 12 | 0.674 | 0.275–1.656 | 0.3902 |
| **SEPT9 mRNA per 1 SD** | 24 | 10 | 0.783 | 0.480–1.278 | 0.3279 |
| **SEPT9 mRNA High vs Low** | 27 | 12 | 0.799 | 0.329–1.943 | 0.6206 |
| **AKR1B1 mRNA per 1 SD** | 24 | 10 | 1.279 | 0.701–2.334 | 0.4221 |
| **AKR1B1 mRNA High vs Low** | 27 | 12 | 2.192 | 0.888–5.409 | 0.0887 |

*Multivariable Cox proportional hazards regression analyses were performed to identify independent predictors of progression-free survival after adjustment for relevant clinicopathological covariates.*
***Abbreviations:*** *CI, confidence interval; HR, hazard ratio; PFS, progression-free survival; SD, standard deviation.*

**Supplementary Table S9. Multivariable Cox proportional hazards regression analyses for overall survival (OS)**

| Marker model | n | Events | HR | 95% CI | p-value |
|---|---|---|---|---|---|
| **SEPT9 methylation (mean CpG) per 1 SD** | 27 | 6 | 1.063 | 0.671–1.684 | 0.7944 |
| **SEPT9 methylation High vs Low** | 27 | 6 | 1.171 | 0.480–2.855 | 0.7282 |
| **AKR1B1 methylation (mean CpG) per 1 SD** | 27 | 6 | 0.953 | 0.581–1.564 | 0.8502 |
| **AKR1B1 methylation High vs Low** | 27 | 6 | 0.962 | 0.398–2.326 | 0.9318 |
| **SEPT9 mRNA per 1 SD** | 24 | 4 | 0.886 | 0.541–1.452 | 0.6313 |
| **SEPT9 mRNA High vs Low** | 27 | 6 | 0.797 | 0.328–1.937 | 0.6162 |
| **AKR1B1 mRNA per 1 SD** | 24 | 4 | 1.071 | 0.629–1.824 | 0.8001 |
| **AKR1B1 mRNA High vs Low** | 27 | 6 | 1.073 | 0.441–2.606 | 0.8771 |

*Multivariable Cox proportional hazards regression analyses were performed to identify independent predictors of overall survival after adjustment for relevant clinicopathological covariates.*
***Abbreviations:*** *CI, confidence interval; HR, hazard ratio; OS, overall survival; SD, standard deviation.*

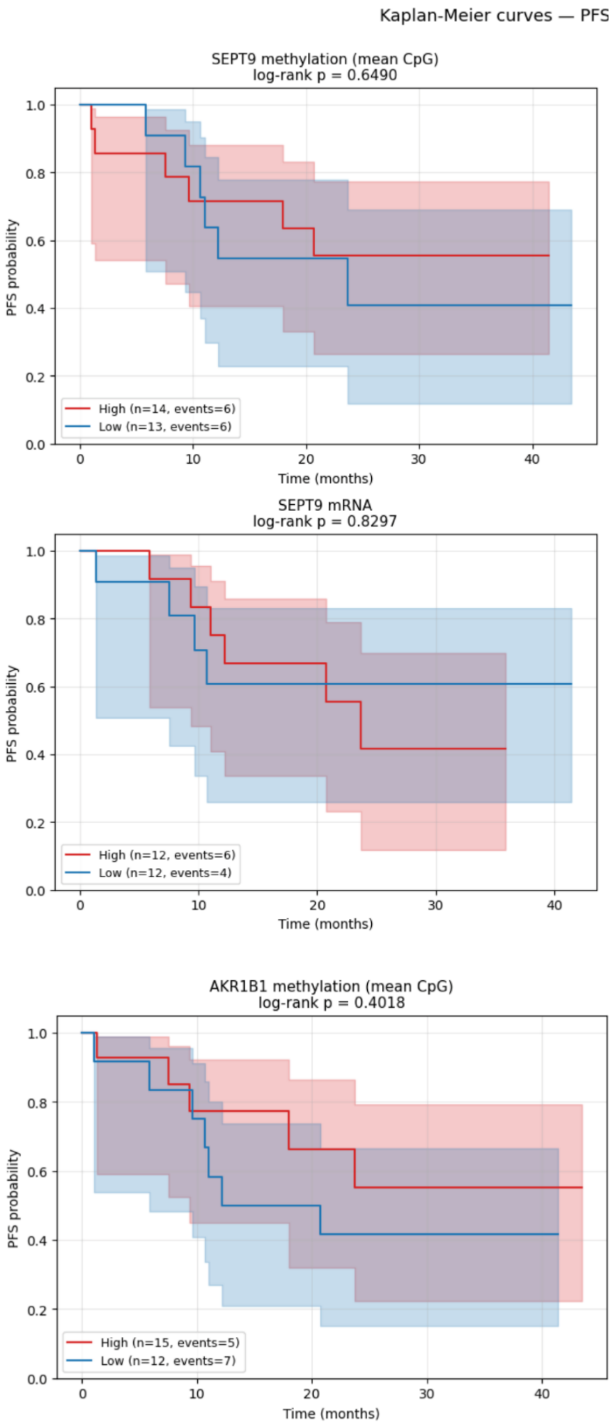


**Supplementary Figure S1. Kaplan–Meier analysis of progression-free survival according to SEPT9 methylation, SEPT9 mRNA expression, and AKR1B1 methylation status.** No significant associations between these molecular markers and progression-free survival were observed (all log-rank $p > 0.05$).

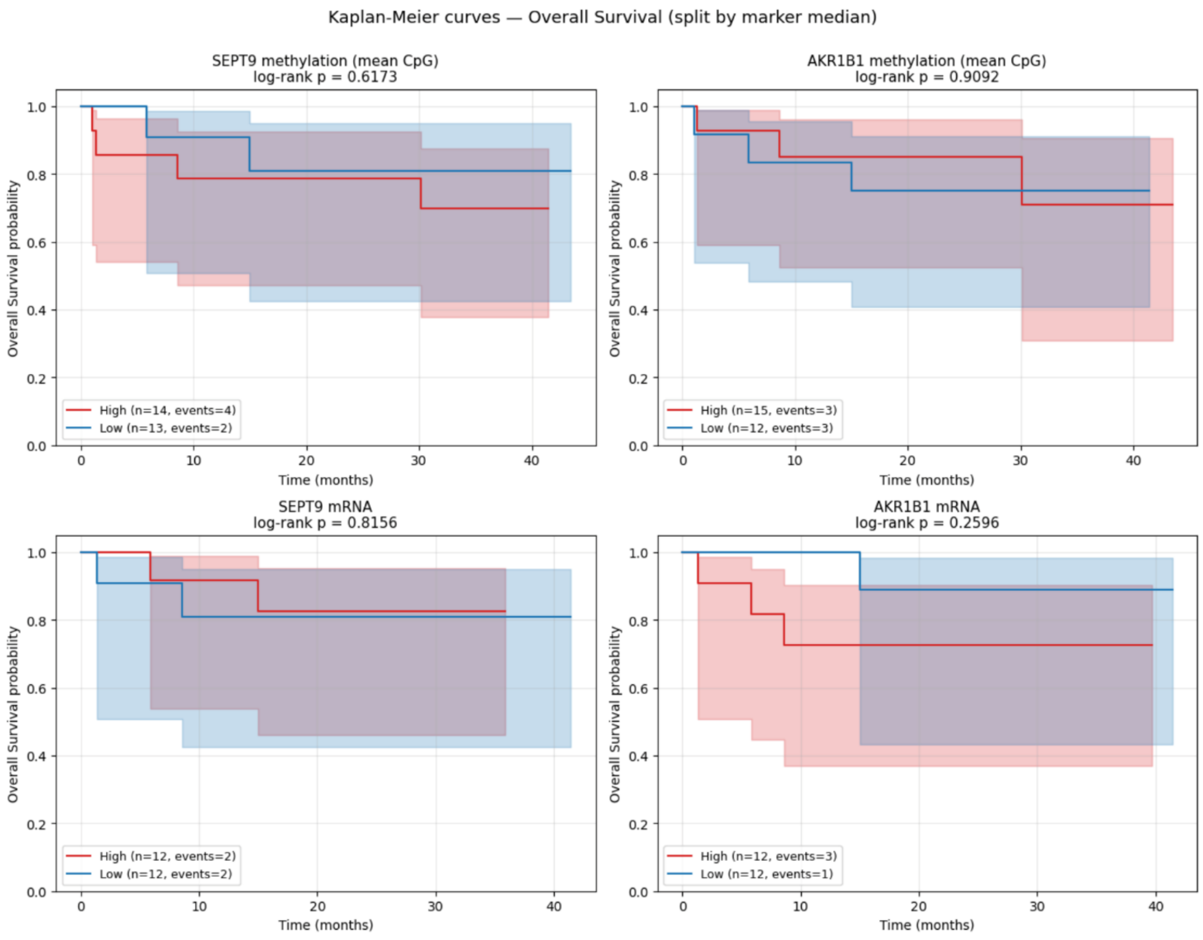


**Supplementary Figure S2. Kaplan–Meier analysis of overall survival according to SEPT9 methylation, SEPT9 mRNA expression, AKR1B1 methylation, and AKR1B1 mRNA expression status.** No significant associations between molecular marker levels and overall survival were identified (all log-rank $p > 0.05$).
